# VEGF as a Paracrine Regulator of Conventional Outflow Facility

**DOI:** 10.1167/iovs.16-20779

**Published:** 2017-03

**Authors:** Ester Reina-Torres, Joanne C. Wen, Katy C. Liu, Guorong Li, Joseph M. Sherwood, Jason Y. H. Chang, Pratap Challa, Cassandra M. Flügel-Koch, W. Daniel Stamer, R. Rand Allingham, Darryl R. Overby

**Affiliations:** 1 Department of Bioengineering, Imperial College London, London, United Kingdom; 2 Department of Ophthalmology, Duke University, Durham, North Carolina, United States; 3 Department of Anatomy II, Friedrich-Alexander University of Erlangen-Nürnberg, Erlangen, Germany

**Keywords:** vascular endothelial growth factor, Schlemm's canal, trabecular meshwork, outflow facility, mouse models

## Abstract

**Purpose:**

Vascular endothelial growth factor (VEGF) regulates microvascular endothelial permeability, and the permeability of Schlemm's canal (SC) endothelium influences conventional aqueous humor outflow. We hypothesize that VEGF signaling regulates outflow facility.

**Methods:**

We measured outflow facility (*C*) in enucleated mouse eyes perfused with VEGF-A_164_a, VEGF-A_165_b, VEGF-D, or inhibitors to VEGF receptor 2 (VEGFR-2). We monitored VEGF-A secretion from human trabecular meshwork (TM) cells by ELISA after 24 hours of static culture or cyclic stretch. We used immunofluorescence microscopy to localize VEGF-A protein within the TM of mice.

**Results:**

VEGF-A_164_a increased *C* in enucleated mouse eyes. Cyclic stretch increased VEGF-A secretion by human TM cells, which corresponded to VEGF-A localization in the TM of mice. Blockade of VEGFR-2 decreased *C*, using either of the inhibitors SU5416 or Ki8751 or the inactive splice variant VEGF-A_165_b. VEGF-D increased *C*, which could be blocked by Ki8751.

**Conclusions:**

VEGF is a paracrine regulator of conventional outflow facility that is secreted by TM cells in response to mechanical stress. VEGF affects facility via VEGFR-2 likely at the level of SC endothelium. Disruption of VEGF signaling in the TM may explain why anti-VEGF therapy is associated with decreased outflow facility and sustained ocular hypertension.

Intraocular pressure (IOP) is determined by the facility of aqueous humor outflow through the conventional outflow pathway. While decreased outflow facility causes IOP elevation in most forms of glaucoma,^1^ the factors controlling outflow facility remain largely unknown. Within the conventional outflow pathway, facility is predominately regulated within the outer trabecular meshwork (TM) and the underlying inner wall endothelium of Schlemm's canal (SC).^2,3^ Aqueous humor likely crosses SC endothelium through micrometer-sized pores,^4–8^ and SC pore density is reduced in glaucoma.^9–11^ As pores may influence outflow facility,^12^ the porosity or permeability of SC endothelium presumably is an important factor controlling outflow facility and, hence, IOP.

Vascular endothelial growth factor (VEGF) is a potent regulator of endothelial permeability.^13^ In vascular endothelia, VEGF induces formation of pore-like fenestrae^14,15^ and disassembly of intercellular junctions.^16,17^ VEGF is secreted by human TM cells in culture, and VEGF has been proposed to regulate the permeability of SC endothelium to affect outflow.^18,19^ The inner wall endothelium of SC expresses all three VEGF receptors (VEGFR), including VEGFR-1 and -2, common to vascular endothelia, and VEGFR-3, which is typical of lymphatic endothelia but absent from vascular endothelia.^20–25^ VEGF increases outflow facility in pigs,^19^ and pigment-epithelium derived factor (PEDF) that functionally antagonizes VEGF decreases outflow facility in mice.^26^ Furthermore, heterozygous deletion of VEGFR-1 and/or VEGFR-2 leads to ocular hypertension and buphthalmia in mice.^22^

Drugs that disrupt VEGF signaling are being used to treat a range of retinal diseases, including neovascular age-related macular degeneration (NVAMD), diabetic macular edema, and retinal vein occlusion. Despite the benefits of anti-VEGF therapy, a number of observational studies have reported sustained ocular hypertension lasting several months or longer in 3% to 11% of patients receiving repeated injections of anti-VEGF.^27–36^ In our companion study^37^ we show that prolonged anti-VEGF therapy is associated with reduced tonographic outflow facility in patients receiving unilateral treatment for NVAMD. These data suggest that disruption of endogenous VEGF signaling inhibits normal outflow function and IOP homeostasis.

We hypothesize that VEGF is a paracrine regulator of conventional outflow facility that is secreted in response to IOP-related mechanical cues. To test this hypothesis, we investigated the effect of different isoforms of VEGF and VEGFR inhibitors on aqueous humor outflow facility, and we localize VEGF protein within the TM of mice. Mice are a valuable animal model for studying outflow because, like primates, mice have a continuous SC and lamellated TM,^38,39^ and mice demonstrate a similar pharmacologic response to compounds that affect outflow facility in humans.^26,40–43^ To mimic the repetitive mechanical stress induced by IOP pulsations within the TM,^44^ we subjected TM cells in culture to cyclic stretch and measured VEGF secretion.

## Materials and Methods

### Ex Vivo Mouse Eye Perfusions

Perfusion of enucleated mouse eyes was used to assess the effect of VEGF or related compounds on pressure-dependent outflow facility. Outflow facility (*C*) was measured in paired contralateral eyes by multilevel constant pressure perfusion using iPerfusion.^45^ All mice were male C57BL/6 (Charles River UK Ltd, Margate, UK) aged between 9 and 13 weeks at the time of perfusion. Mice were fed ad libitum and maintained at 21°C with a 12-hour light/dark cycle. All animals were treated in compliance with the ARVO Statement for the Use of Animals in Ophthalmic and Vision Research under the authority of a UK Home Office Project License.

Eyes were enucleated within 10 minutes of death by cervical dislocation, affixed to a support platform using cyanoacrylate glue, and submerged in a bath of Dulbecco's PBS at 35°C. The perfusion needle and tubing were backfilled with approximately 200 μl of perfusion fluid; either Dulbecco's PBS including divalent cations and 5.5 mM glucose that was passed through a 0.22 μm filter (collectively referred to as “DBG”), or DBG containing the desired concentration of compound. If the compound was solubilized in vehicle, typically DMSO, then an equivalent concentration of vehicle was added to the DBG solution for the contralateral control eye. The anterior chamber was cannulated using a 33-gauge needle mounted on a micromanipulator while visualizing through a stereomicroscope. The eye was pressurized at 8 mm Hg for 45 minutes to allow the eye to equilibrate to the perfusion system and provide time for the drug to permeate through the anterior chamber and outflow pathway. The eye then was perfused over 9 increasing pressure steps from approximately 4 to 20 mm Hg using an actuated reservoir. Intraocular pressure was measured using a differential pressure transducer (PX409; Omegadyne, Sunbury, OH, USA), while the flow rate into the eye was measured using a thermal flow sensor (SLG64-0075; Sensirion, Staefa, Switzerland) as previously described.^45^

**Figure 1:**
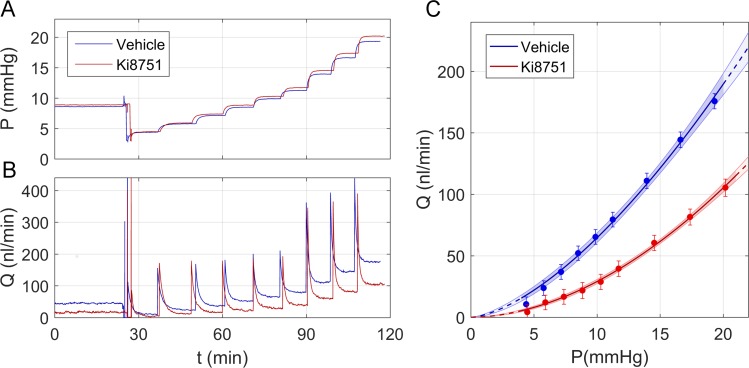
Representative data obtained using the iPerfusion system.^45^ Raw unfiltered data showing the pressure *P* (**A**) and flow rate *Q* (**B**) tracings for a pair of enucleated mouse eyes perfused with either antagonist to VEGFR-2 (1 nM Ki8751 in vehicle, *red tracings*) or vehicle alone (Dulbecco's PBS + 5.5 mM glucose, *blue tracings*). (**C**) The average *Q* versus *P* data from the last 4 minutes of each pressure step using the tracings shown in (**A**) and (**B**) with power law fittings to the data as described in the main text. *Error bars*: 95% CI on the measured value of *Q* for each pressure step accounting for sensor uncertainty. The *shaded blue* and *red regions* indicate the 95% CI on the power law fittings.

For each pressure step, the tracing was considered objectively to have reached steady state once the magnitude of the slope of the ratio of flow rate to pressure, calculated over a 5-minute window, remained consistently below 0.1 nl/min/mm Hg/min for 1 minute. Data were then averaged over the last 4 minutes to calculate the steady state flow rate and pressure values for that step, and the actuated reservoir was automatically elevated to initiate the next pressure step. Pressure steps that did not reach the stability condition defined above were excluded, along with all subsequent steps, and eye pairs with less than 4 stable pressure steps in either eye were excluded. Exclusion rates ranged between 10% and 50% across all experimental sets. For each eye, the stable flow rate (*Q*) and pressure (*P*) values for each step were fit by an empirical power law model of the form *Q = C_r_* (*P/P_r_*)*^β^ P*, where *C_r_* is the facility at a reference pressure *P_r_*, chosen to be 8 mm Hg as the physiologic pressure drop across the outflow pathway,^45^ and *β* describes the nonlinearity in the flow-pressure relationship that represents the pressure dependence of *C*. The difference in facility between contralateral eyes, where one eye received the treatment and the other vehicle control, was the primary readout. The analysis followed the methodology described by Sherwood et al.^45^ to account for various uncertainties associated with the measurement and analysis. Representative perfusion tracings and flow-pressure relationships are provided in Figure 1.

Perfusions included in this study examined the effect of recombinant murine VEGF-A_164_a (Sigma-Aldrich Company Ltd, Dorset, United Kingdom) and VEGF-D (Abcam, Cambridge, United Kingdom) and human VEGF-A_165_b (Abcam), which is an inactive splice variant that lacks the neuropilin binding domain and thereby acts as a competitive inhibitor for the VEGF receptor.^46^ Studies also examined the effect of the VEGFR-2 inhibitors SU5416 (Sigma-Aldrich Company Ltd) and Ki8751 (Selleckchem, Munich, Germany). We also examined the effect on *C* of ranibizumab, an Fab antibody fragment against human VEGF (Novartis, Basel, Switzerland) and a polyclonal antibody against mouse VEGF-A_164_ (AF-493; R&D Biosystems, Minneapolis, MN, USA). Perfusions typically were obtained using contralateral eyes perfused simultaneously using two identical iPerfusion systems, with cannulations occurring within 10 to 30 minutes of enucleation. Exceptions include perfusions with VEGF-A_164_a, VEGF-A_165_b, and SU5416, where contralateral eyes were perfused sequentially because only a single iPerfusion system was available. For these experiments, we randomized the order of perfusion (i.e., whether the treated eye was perfused first or second). During the first perfusion, the second eye was stored in PBS for a maximum of 4 hours at 4°C.

### Human TM Cell Culture, Cell Stretching, and VEGF Secretion

Human TM cells were assayed for VEGF production under static conditions and in response to cyclic stretch. TM cells were isolated and characterized as described previously.^47^ This study included three cell strains aged 64 years (TM89), 72 years (TM121), and an adult donor of unknown age (TM94). TM cells were cultured in a humidified air incubator at 37**°**C and 5% CO_2_ using Dulbecco's modified Eagle's medium (DMEM) supplemented with 10% fetal bovine serum (FBS; Atlanta Biologicals, Flowery Branch, GA, USA), 100 U/ml penicillin, 0.1 mg/ml streptomycin, and 0.29 mg/ml L-glutamine.

For stretching experiments, human TM cells were seeded onto flexible membranes mounted into a 6-well culture plate (BioFlex; Flexcell International Corp., Hillsborough, NC, USA) and grown to confluence under static conditions in DMEM containing 10% FBS. Media was replaced every 2 to 3 days until the cells became confluent, at which time the media was switched to DMEM containing 1% FBS for at least 1 week. Cyclic mechanical stretch (16% peak strain, 1 Hz) was then initiated and continuously applied for 24 hours using a commercial cellular stretching device (FX-5000; Flexcell International Corp.). The applied stretch approximates the predicted strain (7%–33%) if the pressure drop across the TM changes by 2 to 10 mm Hg as occurs during the ocular pulse or saccades,^48^ assuming an elastic modulus of 4 kPa.^49^ Unstretched controls were incubated simultaneously under zero mechanical strain alongside stretched samples. After 24 hours of stretch or static culture, conditioned media was collected and centrifuged at 4000*g* for 3 minutes to pellet cell debris. VEGF-A_165_ concentration was measured in the supernatant using ELISA (Human VEGF Quantikine Kit; R&D Biosystems) and quantified using a microplate reader (SpectraMax M3; Molecular Devices, Sunnyvale, CA, USA).

### Immunofluorescence Labeling

Immunofluorescence was used to label and identify the distribution of VEGF-A within the outflow pathway and limbus of mice. We used two commercially available antibodies: polyclonal rabbit anti-human that cross-reacts with mouse VEGF-A (sc-507; Santa Cruz Biotechnology, Inc., Santa Cruz, CA, USA) and polyclonal goat anti-mouse VEGF-A_164_ (AF-493, R&D Systems). To identify vascular and SC endothelial cells, we dual-labeled with monoclonal rat anti-mouse CD31/PECAM1 (Clone MEC 13.3; BD Biosciences, San Jose, CA, USA). In total, these studies examined 13 eyes from 8 C57BL/6 mice aged 9 to 13 weeks.

The following describes methods used for the polyclonal rabbit antibody applied to 10 eyes from 5 mice. Immediately after enucleation, a pinhole was made through the central cornea, and the eyes were submerged in 4% paraformaldehyde in PBS for 2 hours at room temperature. Eyes were then washed 3 times in PBS for 30 minutes each and bisected at the equator. The lens was removed carefully, and the anterior segments were cut into 4 wedges. Each wedge was frozen in isopentane and embedded into tissue freezing medium (Leica Mikrosysteme Vertrieb GmbH, Wetzlar, Germany). Sagittal sections (10 μm thick) were cut through each wedge using a cryostat (CM3050 S, Leica Mikrosysteme Vertrieb GmbH) and mounted on adhesion slides. After blocking with 1% milk solution for 3 minutes at room temperature, sections were first incubated with rat anti-mouse CD31/PECAM1 diluted 1:100 overnight at 4°C. Following washing in PBS, sections were incubated with goat anti-rat IgG (Alexa Fluor 488; Abcam) diluted 1:1000 for 1 hour at room temperature. Sections were then washed and incubated with polyclonal rabbit anti-VEGF (sc-507, Santa Cruz Biotechnology, Inc.) diluted 1:10 overnight at 4°C. Sections then were washed and incubated with goat anti-rabbit IgG (Alexa Fluor 555; Invitrogen, Life Technologies, Darmstadt, Germany) diluted 1:2000 for 1 hour at room temperature. Sections then were washed and mounted in fluorescent mounting medium (Dako, Biozol, Eching, Germany), and imaged (BZ-9000; Keyence, Neu Isenburg, Germany). Negative controls were processed identically except omitting the primary antibodies. Approximately 144 sections were acquired per eye. Similar methods were used for the second antibody that examined 3 eyes from 3 mice with 10 to 15 sections per eye (See Supplementary Information).

### Statistics

We report the geometric average percent change in facility between contralateral eyes, along with the 95% confidence interval (CI) on the average percent change. Statistical analysis for facility used log-transformed data, with the weighted *t*-test used to calculate significance, as described previously.^45^ A 1-way repeated measures ANOVA on log-transformed concentration values was used to compare the VEGF-A expression by TM cells.

## Results

### VEGF-A Increases Aqueous Humor Outflow Facility

**Figure 2:**
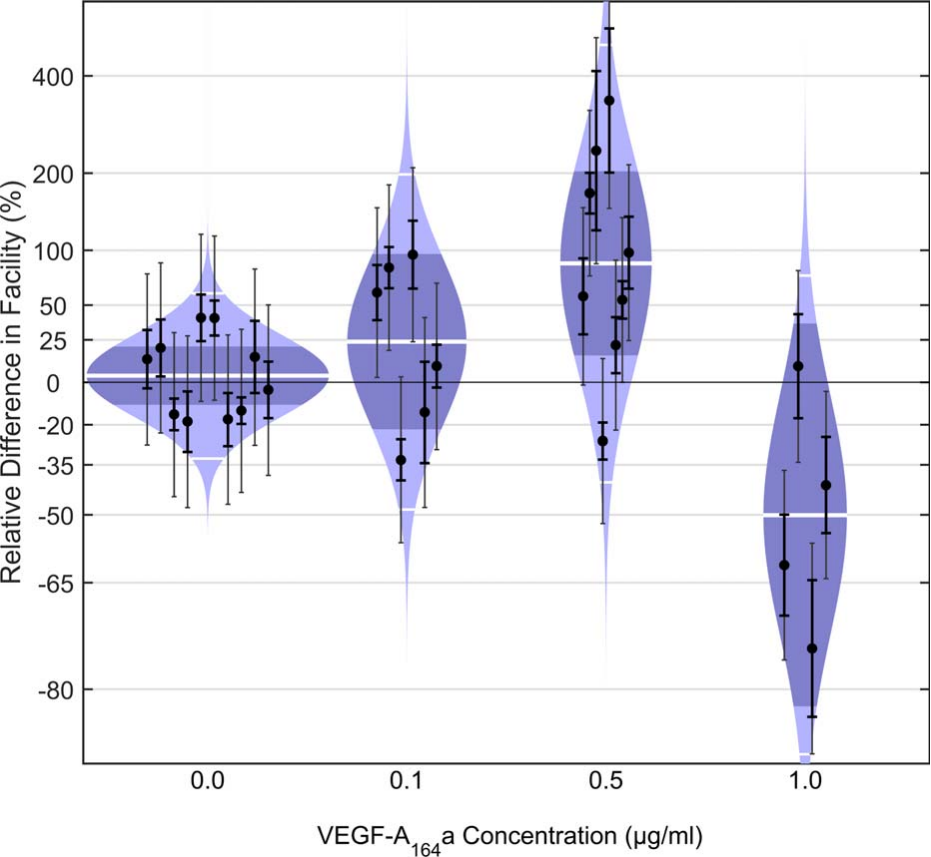
VEGF-A_164_a increases *C* in enucleated mouse eyes. Modified violin or “cello” plots showing the difference in facility for enucleated C57BL/6 mouse eyes perfused with varying concentrations of VEGF-A_164_a relative to contralateral eyes perfused with vehicle. Paired eyes perfused with vehicle in both eyes (0 μg/ml) indicate the expected range of variability between untreated contralateral eyes, which showed an average difference in facility of 4% that was not statistically significant (*P* = 0.62, *n* = 10 pairs, weighted *t*-test). At a low concentration of 0.1 μg/ml VEGF-A_164_a, the average difference in facility was 24% (CI: −22, 96%; *P* = 0.29, *n* = 6, weighted *t*-test). At an intermediate concentration of 0.5 μg/ml VEGF-A_164_a, facility was significantly increased on average by 87% (CI: 15, 203%; *P* = 0.02, *n* = 8). A higher concentration of 1.0 μg/ml VEGF-A_164_a decreased facility by 50% on average (CI: −82, 36%; *P* = 0.11, *n* = 4). Data points represent the measured relative facility difference of the treated eye with respect to the contralateral untreated eye for individual pairs. *Thick error bars* represent the 95% CI on the measured relative difference in facility, while the *thin error bars* represent the additional uncertainty due to the variability between untreated contralateral eyes measured from a previous study.^45^ The colored regions represent the log-normal distribution that best describes the data, while the *thick white central lines* indicate the geometric means. The *dark shaded regions* indicate the 95% CI on the geometric mean, and the *outer thin white lines* indicate the range that encompasses approximately 95% of the measured data.

We first examined the effect of exogenous VEGF-A_164_a on outflow facility in enucleated eyes from C57BL/6 mice using the iPerfusion system.^45^ This isoform was chosen because it typically is the most prevalent isoform and retains binding affinity to VEGFR-2.^50^ We compared *C* between paired eyes perfused with murine VEGF-A_164_a, and their contralateral control eyes perfused with vehicle alone, over 4 different concentrations (0, 0.1, 0.5, 1.0 μg/ml, equivalent to 0, 3, 13, and 26 nM; Fig. 2). In control experiments where both eyes were perfused without VEGF-A_164_a, there was no observed difference in *C* between paired eyes (mean 4%; CI: −11, 21%; *P* = 0.62; *n* = 10 pairs; weighted *t*-test). At the lowest concentration of 0.1 μg/ml VEGF-A_164_a, the average difference in *C* was 24% (CI: −22, 96%), which was not significantly different from zero (*P* = 0.29, *n* = 6). At an intermediate concentration of 0.5 μg/ml, *C* was significantly increased on average by 87% (CI: 15, 203%; *P* = 0.02, *n* = 8). An increase in *C* was not observed, however, at a higher concentration of 1 μg/ml; the average difference being −50% (CI: −82, 36%; *P* = 0.11, *n* = 4). These data indicate that VEGF-A_164_a increases outflow facility at a concentration of approximately 0.5 μg/ml.

### VEGF-A Is Expressed Endogenously Within the Conventional Outflow Pathway

**Figure 3:**
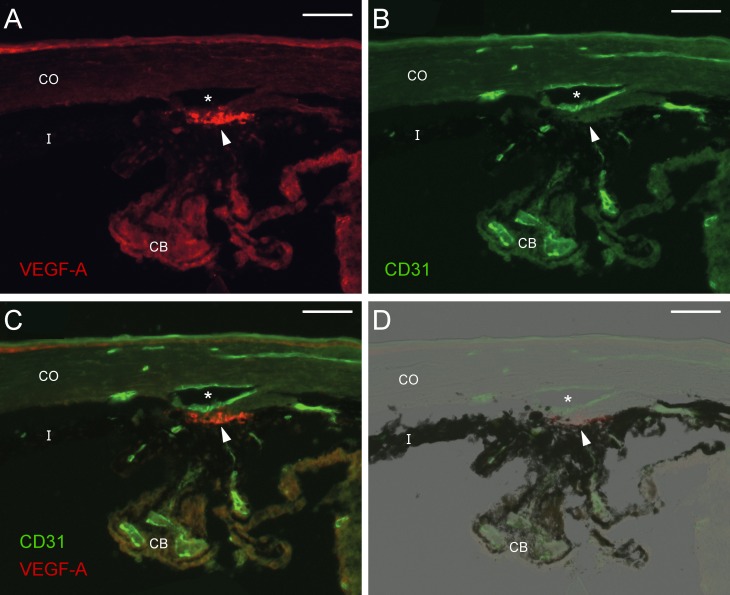
VEGF-A is present within the murine trabecular meshwork. Immunohistochemistry of the limbus in C57BL/6 mice showing the localization of VEGF-A (**A**, *red*, sc-507), CD31/PECAM-1 (**B**, *green*), the merged image (**C**) and merged with bright field (**D**). Intense VEGF-A labeling is present within the trabecular meshwork (*arrowhead*) near the inner wall endothelium of SC (*canal lumen) that expresses CD31. Weaker VEGF-A labeling is present in the corneal epithelium, ciliary epithelium and surrounding CD31-positive vessels in the ciliary body (CB). *Scale bars*: 50 μm. CO, cornea; I, iris.

We immunolabeled the conventional outflow pathway of mice to determine whether VEGF-A is present within the TM in situ, where it may influence outflow facility. As visualized using two different antibodies, intense labeling was observed in the TM, particularly in the innermost lamellated region. Single cells were stained in some regions of the juxtacanicular tissue (JCT), whereas most of the JCT appeared unstained, indicating lower levels of VEGF-A expression in the JCT compared to the innermost TM (Fig. 3; Supplementary Fig. S1). VEGF-A labeling also was present in the corneal epithelium, with weaker labeling observed along the ciliary epithelium and surrounding the CD31-positive vessels in the ciliary processes. Similar weak labeling was present in the ciliary body. Control sections that were treated with secondary antibody alone, showed no staining (Supplementary Fig. S1).

### VEGF-A Is Secreted by TM Cells in Response to Mechanical Stretch

**Figure 4:**
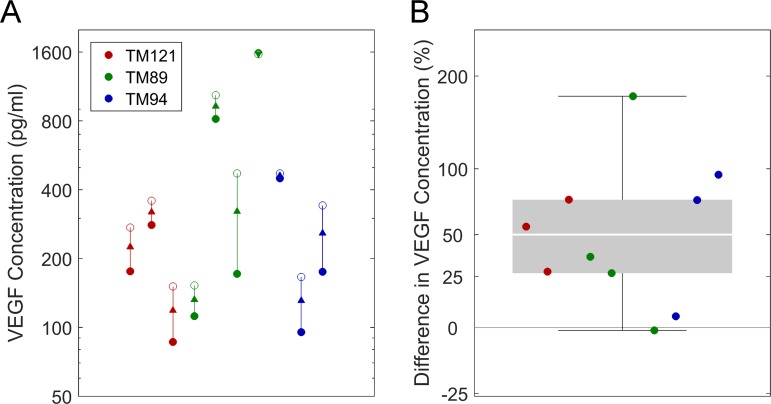
Cyclic stretch promotes VEGF-A secretion from human TM cells. VEGF-A concentration in conditioned medium from human TM cells in static culture (**A**, *filled symbols*) versus 16% cyclic mechanical stretch at 1 cycle per second for 24 hours (*open symbols*). Data points show replicate experiments from 3 different human TM cell strains (TM121 in *red n* = 3, TM89 in *green n* = 4, TM94 in *blue n* = 3) with and without stretch. *Arrowheads* indicate the direction of change in response to stretch. (**B**) The relative increase in VEGF-A concentration in conditioned medium from stretched versus static TM cells based on the data shown in (**A**). Stretch increases VEGF-A production on average by 50% (*P* = 0.008, *n* = 3 cell strains, 1-way repeated measured ANOVA). *Shaded box* indicates the interquartile range. *Error bars*: indicate the range, and the *thick white line* indicates the median.

We then examined the production of VEGF-A by human TM cells in static culture and in response to cyclic mechanical stretch. The latter mimics the in vivo environment where mechanical stimulation is imposed on the TM from continuous pressure pulsations associated with the ocular pulse or saccades.^48^ After 24 hours of static culture, the average VEGF-A concentration in conditioned medium was 246 (CI: 124, 487) pg/ml (mean, 95% CI; *n* = 10, 3 cell strains, 3–4 samples per strain). The average VEGF-A concentration was significantly increased to 369 (CI: 210, 648) pg/ml after 24 hours of cyclic stretch (Fig. 4A), corresponding to a mean relative increase of 50% (*P* = 0.008, *n* = 10, 3 cell strains, 3–4 samples per strain, 1-way repeated measures ANOVA; Fig. 4B). No difference was observed in VEGF-A expression between cell strains (*P* = 0.99).

### VEGFR-2 Mediates the Effects of Endogenous VEGF-A on Outflow Facility

**Figure 5:**
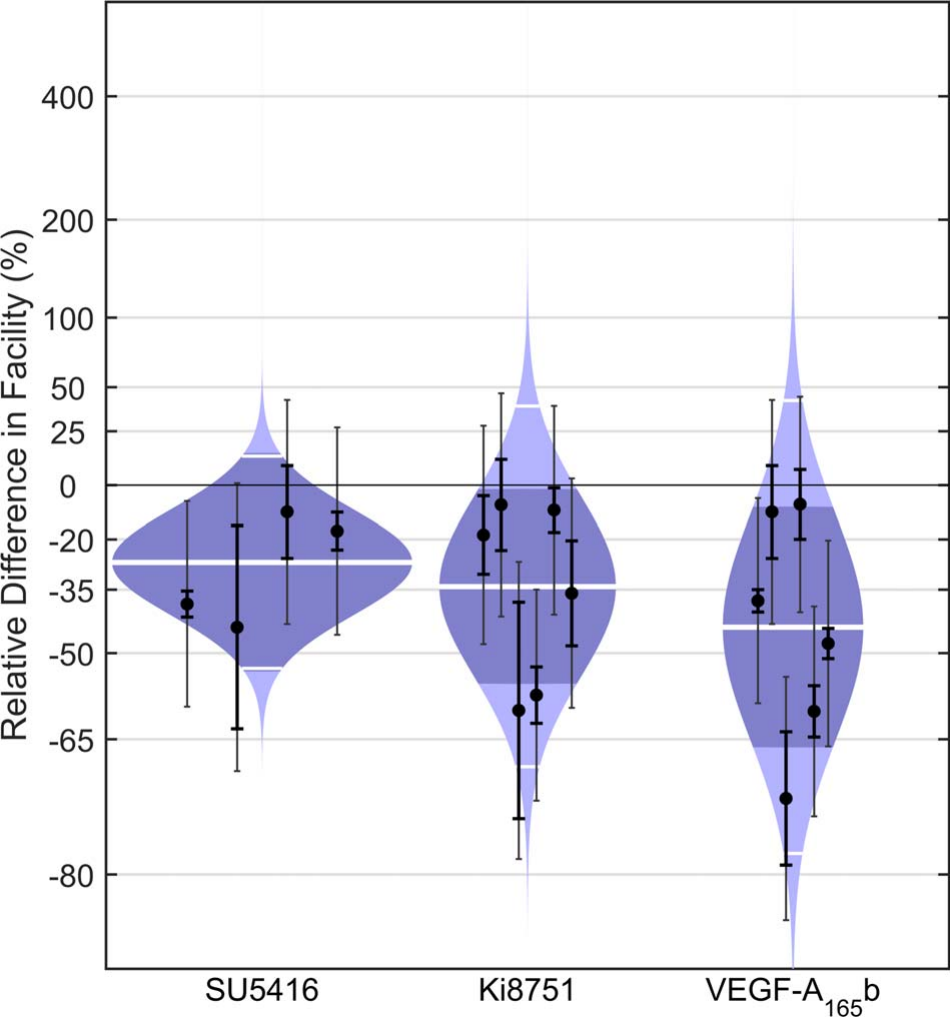
Antagonists to VEGFR-2 decrease outflow facility in enucleated mouse eyes. Cello plots showing the relative difference in *C* between contralateral eyes of C57BL/6 mice perfused with or without 3 μM SU5416, 1 nM Ki8751, or 0.5 μg/ml human VEGF-A_165_b. Ki8751 (*P* = 0.04, *n* = 6, weighted *t*-test) and VEGF-A_165_b (*P* = 0.03, *n* = 6) reduced facility by 34% (CI: −56, −2%) and 44% (CI: −66, −8%) on average, respectively, while SU5416 reduced facility on average by 27% (CI: −54, 14%) but did not achieve significance (*P* = 0.10, *n* = 4). Data points represent the relative facility difference of a treated eye with respect to its contralateral untreated eye for individual pairs. The *thick white lines* represent the geometric mean of the relative difference for each group. The *remaining symbols* are as defined in Figure 2.

To determine whether VEGF-A produced by the TM influences outflow facility through VEGFR-2, we perfused enucleated mouse eyes with VEGFR antagonists SU5416 or Ki8751 in the absence of exogenous VEGF (Fig. 5). In initial studies with 3 μM SU5416, a moderately selective antagonist to VEGFR-2 with an IC_50_ of 1 μM,^51–53^ we observed a decrease in facility in all eyes with an average difference in *C* of −27% (CI: −54, 14%; *P* = 0.10, *n* = 4). We then investigated the response to a more selective antagonist of VEGFR-2, Ki8751, which has an IC_50_ of 0.9 nM.^54^ In response to 1 nM Ki8751, *C* decreased with an average difference of −34% (CI: −56, −2%; *P* = 0.04, *n* = 6). As an alternative approach to inhibit endogenous VEGF signaling, we perfused enucleated mouse eyes with human VEGF-A_165_b, an alternative splice variant of VEGF-A that acts as a competitive inhibitor of VEGF receptor activity.^46^ VEGF-A_165_b lacks the neuropilin-binding domain required for VEGF receptor activation, while retaining its ligand-binding domain to VEGFR-1 and -2.^55–57^ In the presence of 0.5 μg/ml VEGF-A_165_b, *C* was decreased with an average difference of −44% (CI: −66, −8%; *P* = 0.03; *n* = 6). These data demonstrated, using three different agents, that blockade of VEGFR-2 decreases *C* in mice, presumably by inhibiting endogenous VEGF signaling in the TM.

### VEGFR-2 Mediates the Effects of VEGF-D on Outflow Facility

**Figure 6:**
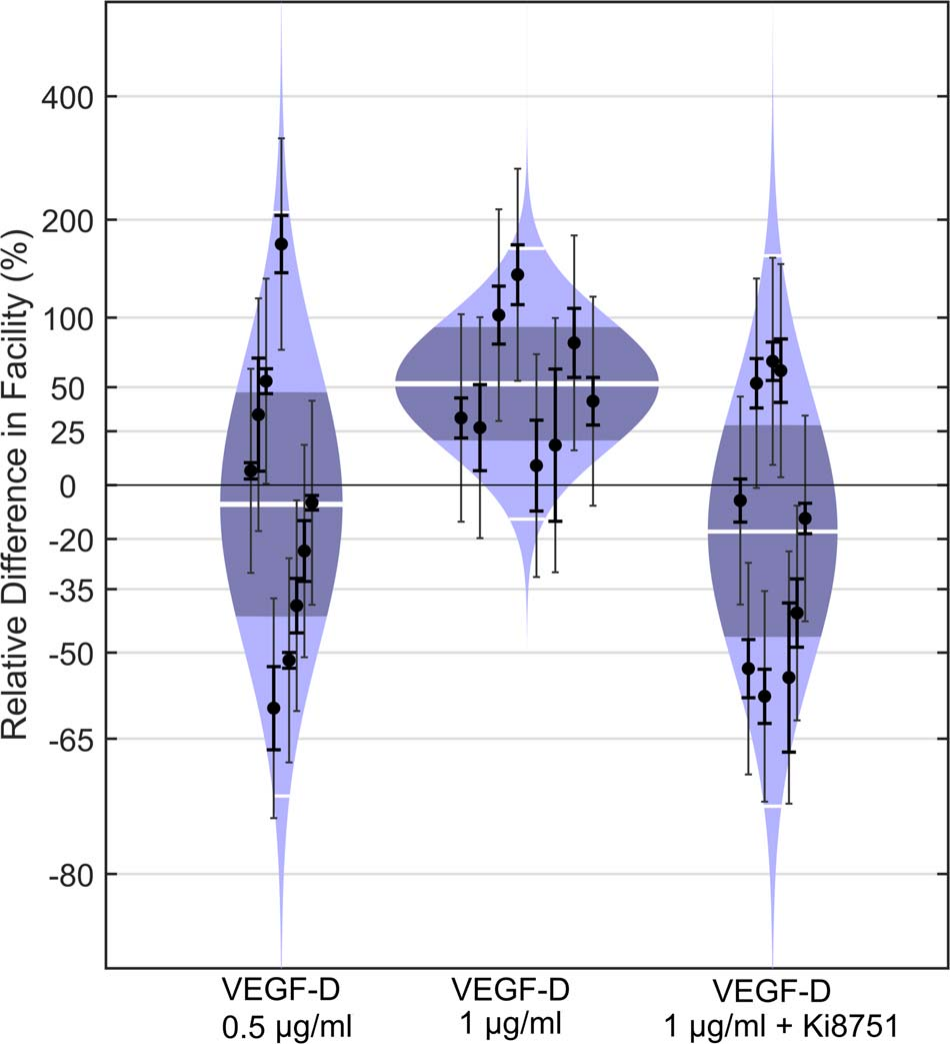
VEGF-D increases outflow facility via VEGFR-2 in enucleated mouse eyes. Cello plots showing the relative difference in *C* between contralateral eyes of C57BL/6 mice perfused with or without VEGF-D at 0.5 or 1.0 μg/ml. The average facility difference was 52% in response to 1.0 μg/ml VEGF-D (*P* = 0.004, *n* = 8, weighted *t*-test), while 0.5 μg/ml VEGF-D had no detectible effect on facility with an average difference of −8% (*P* = 0.70, *n* = 9). No facility difference was observed in response to 1.0 μg/ml VEGF-D in the presence of 1 nM Ki8751 compared to the contralateral eye perfused with Ki8751 alone (*P* = 0.35, *n* = 9), indicating that the facility-increasing effect of VEGF-D is mediated via VEGFR-2. Data points represent the relative facility difference of a treated eye with respect to its contralateral untreated eye for individual pairs. The *thick white lines* represent the geometric mean of the relative difference for each group. The remaining symbols are as defined in Figure 2.

To determine whether the activation of VEGFR-2 is specific to VEGF-A, we measured outflow facility in mice following perfusion with VEGF-D. VEGF-D is an endogenous ligand for VEGFR-2 and -3, which typically are expressed by lymphatic endothelia^58,59^ as well as SC endothelium, along with VEGFR-1.^20–25^ Perfusion with 1 μg/ml murine VEGF-D significantly increased *C* in all eyes on average by 52% (CI: 20, 92%; *P* = 0.004, *n* = 8), while 0.5 μg/ml VEGF-D had no detectable effect on *C* with an average difference of −8% (CI: −42, 47%; *P* = 0.70, *n* = 9; Fig. 6). The selective antagonist to VEGFR-2, Ki8751 (1 nM), blocked the effect of 1 μg/ml VEGF-D compared to contralateral eyes perfused with 1 nM Ki8751 alone, with an average facility difference of −17% (CI: −47, 28; *P* = 0.35, *n* = 9). These data demonstrated that modulation of outflow facility via VEGFR-2 is not specific to VEGF-A.

### Acute Sequestration of VEGF-A Does Not Significantly Affect Outflow Facility

To examine whether sequestration of VEGF-A affects outflow facility over acute time scales, we perfused mouse eyes with 0.14 mg/ml ranibizumab, a dose that is equivalent to that used to treat patients with NVAMD, or vehicle. Ranibizumab had no measurable effect on *C* with an average difference of −5% (CI: −25, 21%; *P* = 0.58, *n* = 5; Supplementary Fig. S2). We reasoned that the absence of an observable facility effect might be attributable to the reported low binding affinity of ranibizumab to the murine homolog of VEGF-A.^60,61^ To address this possibility, we perfused eyes with 0.14 mg/ml of a polyclonal anti-VEGF antibody that is selective and reportedly neutralizing for murine VEGF-A_164_, although it may not distinguish between VEGF-A_164_a and VEGF-A_164_b. These data also showed no apparent effect on *C* with an average facility difference of 3% (CI: −40, 64%; *P* = 0.85, *n* = 4; Supplementary Fig. S2). Therefore, acute exposure to anti-VEGF compounds may be insufficient to affect outflow facility over relatively short time scales (corresponding to the ∼2-hour duration of the perfusion), suggesting that prolonged exposure to VEGF-sequestering compounds may be necessary to significantly affect outflow function, possibly due to homeostatic compensation or to a sufficiently large intraocular reservoir of endogenous VEGF-A.

## Discussion

**Figure 7:**
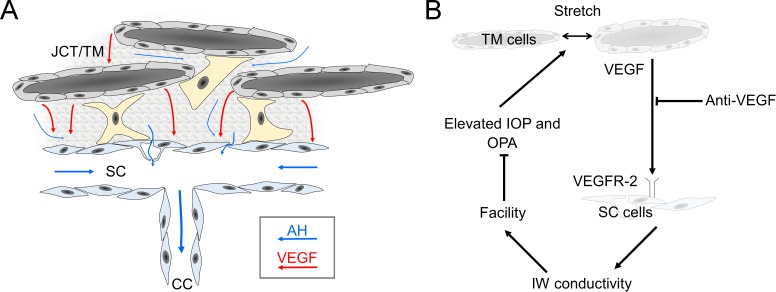
VEGF is a paracrine regulator of outflow facility that may be involved in IOP homeostasis. (**A**) VEGF secretion by TM cells may reach the inner wall (IW) of SC via either diffusion or advection due to aqueous humor (AH) outflow. (**B**) VEGF release in the TM increases in response to cyclic stretch that depends on ocular pulse amplitude (OPA) that increases with IOP. VEGF modulates facility via VEGFR-2 presumably at the level of SC endothelium to increase IW conductivity and outflow facility. The facility increase of VEGF opposes IOP and OPA elevation, and may thereby contribute to IOP homeostasis. Anti-VEGF therapy may disrupt VEGF signaling in the TM to cause ocular hypertension, as examined in our companion study.^37^

In this study, we demonstrate that VEGF modulates outflow facility in mice. We showed that human TM cells secrete VEGF-A in culture, consistent with earlier observations,^18^ and that VEGF-A is present within the murine TM. Pharmacologic blockade of VEGFR-2, which is expressed by SC endothelium,^21,22,24,25^ decreases outflow facility in mice, presumably by suppressing endogenous VEGF signaling within the TM/SC. These studies suggested a mechanism by which VEGF production within the TM acts as a paracrine signal to modulate outflow facility, presumably by altering the permeability of SC endothelium (Fig. 7).

VEGF is a potent regulator of endothelial permeability.^13^ VEGF widens paracellular spaces and induces formation of small transcellular pores known as fenestrae in otherwise continuous endothelia of some vascular beds.^62^ Fenestrated endothelia typically are supported by paracrine VEGF expression from neighboring cells,^63^ and disruption of local VEGF expression often leads to loss of endothelial fenestrations.^64^ Like fenestrated endothelia, the inner wall of SC contains micrometer-sized pores as well as smaller “mini-pores” that have a similar ultrastructure to diaphragmed fenestrae.^5,65^ VEGF secreted by TM cells likely reaches and acts on the inner wall, which uniquely expresses all three VEGFRs,^21,22,24,25^ to potentially regulate pore formation.^18^ This provides a putative mechanism by which VEGF may modulate the hydraulic conductivity of SC endothelium to influence outflow facility.

The influence of VEGF on outflow facility mimics the VEGF response on vascular endothelia, where permeability effects are mediated via VEGFR-2.^66–68^ Likewise, in the outflow pathway, ligands for VEGFR-2, such as VEGF-A and VEGF-D, increase outflow facility while antagonists to VEGFR-2 decrease facility or block the effect of exogenous VEGF-D. Hence, as for vascular endothelia, VEGFR-2 appears to mediate the effects of VEGF on outflow facility, presumably by regulating the hydraulic conductivity of SC inner wall. This is consistent with recent data showing that VEGFR-2 mediates the effect of VEGF-A_121_ on the barrier function of cultured SC cells from nonhuman primates.^19^ However, despite its vascular origins,^24,69^ SC inner wall exhibits characteristics of lymphatic endothelia,^20^ namely expression of VEGFR-3.^25^ The unique lymphatic/vascular dual nature of SC differentiates it from other vasculature within the eye. Therefore, exploiting VEGFR-3 to target SC inner wall is an appealing strategy to affect outflow in glaucoma, as this could potentially minimize off-target effects on other endothelia. However, there are no known isoforms of VEGF that are selective for VEGFR-3, and VEGF-C and VEGF-D bind VEGFR-2 as well as VEGFR-3 (for review on VEGFRs activation and signaling see the report of Simons et al.^70^). In this study, we chose to examine VEGF-D because, in addition to signaling via VEGFR-3, it also inhibits 15-hydroxyprostaglandin dehydrogenase,^71^ resulting in higher prostaglandin availability that may improve TM outflow.^72,73^ However, despite VEGF-D increasing facility, its effects appeared to be entirely mediated via VEGFR-2. Although this observation may be attributable to the relatively short experimental exposure time or to differential sensitivity for VEGF concentration between receptors, these data suggested that signaling via VEGFR-3 is unlikely to affect outflow facility. VEGF-Rs also can be activated in a VEGF-independent manner, for example in response to oxidative stress^74^ or upon interaction with integrins,^75^ which would contribute to additional receptor activity. Further work is necessary to clarify the exact role of the different VEGF-Rs, their coreceptors, such as neuropilin, and other VEGF isoforms on outflow facility.

The increase in VEGF-A production by TM cells in response to cyclic stretch suggests that VEGF-A expression may be regulated in part by mechanical forces associated with IOP. IOP is not static in a living eye, but experiences continuous oscillations of 2 to 3 mm Hg due to the ocular pulse.^48^ Larger IOP changes of up to 10 mm Hg are expected due to blinking or saccades.^48^ The ocular pulse imposes cyclic stretch within the TM^44^ and the magnitude of the ocular pulse increases with IOP.^76^ Thus, cyclic stretch is a physiologic stressor, the magnitude of which depends upon IOP, that acts continuously on TM cells in vivo. Contraction of the ciliary muscle may apply additional mechanical stimulation to TM cells via anterior ciliary muscle tendons that insert into the TM.^39,43,77^ We thereby propose that stretch-induced VEGF production may provide a mechanosensitive feedback signal within the TM to modulate outflow facility for IOP homeostasis. Similar mechanisms have been proposed for metalloproteinases^78^ and ATP.^79^ Any stretch-induced VEGF production would be superimposed on background levels already present within aqueous humor (reported to be between 15 and 533 pg/ml^80–88^). VEGF levels also are subject to binding to heparin sulfate proteoglycans and may be affected by several factors, including TGF-β2,^18^ BMP-7,^18^ hypoxia,^89^ as well as additional VEGF secretion by any macrophages^90^ that may be present within the TM.^91,92^

### Clinical Implications

Antiangiogenic antibodies or antibody fragments that bind and sequester VEGF are used to treat a variety of retinal vascular disorders, including NVAMD, diabetic macular edema and retinal vein occlusion. A small but significant portion of patients (3%–11%) receiving intravitreal anti-VEGF experience sustained ocular hypertension.^28,29,32,93^ Proposed mechanisms for ocular hypertension include obstruction of the TM by protein aggregates^94^ or foreign particles^35^ or by damage to the outflow pathway cells.^34^ However, following intravitreal injection, anti-VEGF antibodies permeate the anterior chamber,^86^ reduce the levels of VEGF-A within aqueous humor,^86,88^ and enter the TM and SC by bulk outflow.^95^ Within the TM, anti-VEGF antibodies would presumably interfere with VEGF signaling to disrupt outflow, similar to facility decrease observed in response to pharmacological blockade of VEGFR-2. Our perfusion studies with anti-VEGF antibodies suggest that acute antibody exposure is insufficient to affect outflow function, but this does not exclude the possibility for outflow disruption following long-term exposure. Based on these data, we hypothesize that prolonged exposure to anti-VEGF therapy induces sustained ocular hypertension by disrupting endogenous VEGF signaling in the TM/SC that is involved in IOP homeostasis.

Consistent with this hypothesis, in our companion study^37^ we show that intravitreal anti-VEGF therapy reduces tonographic outflow facility in some patients. Interestingly, patients with the largest facility reduction were those who exhibited ocular hypertension independent of anti-VEGF therapy. These data suggested that patients with compromised aqueous humor dynamics, in many cases due to reduced outflow facility, are more susceptible to outflow disruption induced by anti-VEGF therapy. In other words, patients with a dysfunctional TM are at greater risk for further TM damage, IOP elevation, and vision loss caused by glaucomatous optic nerve damage. Patients who are most at risk are those who are ocular hypertensive (> = 21 mm Hg) at the start of anti-VEGF therapy or in whom ocular hypertension develops during therapy. As elevated IOP is a major risk factor for glaucoma, screening patients for elevated IOP before initiating anti-VEGF therapy can help identify patients who should be monitored more closely or who may require glaucoma therapy. This is particularly important since patients receiving anti-VEGF therapy, who have lost vision due to retinal disease, are at risk of additional vision loss caused by optic nerve damage.

In conclusion, VEGF is a paracrine regulator of outflow facility that is likely involved in IOP homeostasis as summarized by the model shown in Figure 7. VEGF is secreted by TM cells in response to IOP-dependent mechanical cues to affect outflow facility, presumably by modulating the hydraulic conductivity of SC inner wall. Differential isoforms of VEGF provoke bidirectional changes in outflow facility via VEGFR-2, consistent with effects of VEGF on microvascular endothelial permeability and the vascular origins of SC. Disrupting VEGF signaling in the TM, as may occur during intravitreal anti-VEGF therapy, may contribute to TM outflow dysfunction and sustained ocular hypertension. These data reinforced the notion that the hydraulic conductivity of SC inner wall is a key regulator of outflow facility and identify VEGF as a potential regulator of IOP homeostasis.

## Supplementary Material

Supplement 1

## References

[1] Grant WM. Clinical measurements of aqueous outflow. *Am J Ophthalmol*. 1951; 34: 1603–1605. 14885362

[2] Lütjen-Drecoll E. Structural factors influencing outflow facility and its changeability under drugs. A study in *Macaca arctoides*. *Invest Ophthalmol Vis Sci*. 1973; 12: 280–294. 4144361

[3] Mäepea O, Bill A. Pressures in the juxtacanalicular tissue and Schlemm's canal in monkeys. *Exp Eye Res*. 1992; 54: 879–883. 1521580 10.1016/0014-4835(92)90151-h

[4] Holmberg A. The fine structure of the inner wall of Schlemm's canal. *AMA Arch Ophthalmol*. 1959; 62: 956–958.

[5] Inomata H, Smelser GK, Bill A. Aqueous humor pathways through trabecular meshwork and into Schlemm's canal in cynomolgus monkey (Macaca Irus). *Am J Ophthalmol*. 1972; 73: 760–790. 4623937 10.1016/0002-9394(72)90394-7

[6] Grierson I, Lee WR, Moseley H, Abraham S. The trabecular wall of Schlemm's canal: a study of the effects of pilocarpine by scanning electron microscopy. *Br J Ophthalmol*. 1979; 63: 9–16. 760777 10.1136/bjo.63.1.9 PMC1043378

[7] Ethier CR, Coloma FM, Sit AJ, Johnson M. Two pore types in the inner-wall endothelium of Schlemm's canal. *Invest Ophthalmol Vis Sci*. 1998; 39: 2041–2048. 9761282

[8] Braakman ST, Read AT, Chan DW, Ethier CR, Overby DR. Colocalization of outflow segmentation and pores along the inner wall of Schlemm's canal. *Exp Eye Res*. 2015; 130: 87–96. 25450060 10.1016/j.exer.2014.11.008 PMC4305530

[9] Allingham RR, Dekater AW, Ethier CR, The relationship between pore density and outflow facility in human eyes. *Invest Ophthalmol Vis Sci*. 1992; 33: 1661–1669. 1559766

[10] Johnson M, Chan D, Reed AT, The pore density in the inner wall endothelium of Schlemm's canal of glaucomatous eyes. *Invest Ophthalmol Vis Sci*. 2002; 43: 2950–2955. 12202514

[11] Overby DR, Zhou EH, Vargas-Pinto R, Altered mechanobiology of Schlemm's canal endothelial cells in glaucoma. *Proc Natl Acad Sci U S A*. 2014; 111: 13876–13881. 25201985 10.1073/pnas.1410602111 PMC4183270

[12] Johnson M, Shapiro A, Ethier CR, Kamm RD. Modulation of outflow resistance by the pores of the inner wall endothelium. *Invest Ophthalmol Vis Sci*. 1992; 33: 1670–1675. 1559767

[13] Senger DR, Connolly DT, Van de Water L, Feder J, Dvorak HF. Purification and NH2-terminal amino acid sequence of guinea pig tumor-secreted vascular permeability factor. *Cancer Res*. 1990; 50: 1774–1778. 2155059

[14] Esser S, Wolburg K, Wolburg H, Vascular endothelial growth factor induces endothelial fenestrations in vitro. *J Cell Biol*. 1998; 140: 947–959. 9472045 10.1083/jcb.140.4.947 PMC2141756

[15] Satchell SC, Braet F. Glomerular endothelial cell fenestrations: an integral component of the glomerular filtration barrier. *Am J Physiol Renal Physiol*. 2009; 296: F947–F956. 19129259 10.1152/ajprenal.90601.2008 PMC2681366

[16] Gavard J, Gutkind JS. VEGF controls endothelial-cell permeability by promoting the beta-arrestin-dependent endocytosis of VE-cadherin. *Nat Cell Biol*. 2006; 8: 1223–1234. 17060906 10.1038/ncb1486

[17] Monaghan-Benson E, Burridge K. The regulation of vascular endothelial growth factor-induced microvascular permeability requires Rac and reactive oxygen species. *J Biol Chem*. 2009; 284: 25602–25611. 19633358 10.1074/jbc.M109.009894 PMC2757962

[18] Fuchshofer R, Stephan DA, Russell P, Tamm ER. Gene expression profiling of TGFbeta2- and/or BMP7-treated trabecular meshwork cells: identification of Smad7 as a critical inhibitor of TGF-beta2 signaling. *Exp Eye Res*. 2009; 88: 1020–1032. 19450457 10.1016/j.exer.2009.01.002 PMC3014319

[19] Fujimoto T, Inoue T, Maki K, Inoue-Mochita M, Tanihara H. Vascular endothelial growth factor-A increases the aqueous humor outflow facility. *PLoS One*. 2016; 11: e0161332. 27584577 10.1371/journal.pone.0161332 PMC5008796

[20] Ramos RF, Hoying JB, Witte MH, Stamer WD. Schlemm's canal endothelia, lymphatic, or blood vasculature? *J Glaucoma*. 2007; 16: 391–405. 17571003 10.1097/IJG.0b013e3180654ac6

[21] Perkumas KM, Stamer WD. Protein markers and differentiation in culture for Schlemm's canal endothelial cells. *Exp Eye Res*. 2012; 96: 82–87. 22210126 10.1016/j.exer.2011.12.017 PMC3296889

[22] Sano K, Katsuta O, Shirae S, Flt1 and Flk1 mediate regulation of intraocular pressure and their double heterozygosity causes the buphthalmia in mice. *Biochem Biophys Res Commun*. 2012; 420: 422–427. 22426483 10.1016/j.bbrc.2012.03.011

[23] Karpinich NO, Caron KM. Schlemm's canal: more than meets the eye, lymphatics in disguise. *J Clin Invest*. 2014; 124: 3701–3703. 25061871 10.1172/JCI77507 PMC4151199

[24] Kizhatil K, Ryan M, Marchant JK, Henrich S, John SW. Schlemm's canal is a unique vessel with a combination of blood vascular and lymphatic phenotypes that forms by a novel developmental process. *PLoS Biol*. 2014; 12: e1001912. 25051267 10.1371/journal.pbio.1001912 PMC4106723

[25] Aspelund A, Tammela T, Antila S, The Schlemm's canal is a VEGF-C/VEGFR-3-responsive lymphatic-like vessel. *J Clin Invest*. 2014; 124: 3975–3986. 25061878 10.1172/JCI75395 PMC4153703

[26] Rogers ME, Navarro ID, Perkumas KM, Pigment epithelium-derived factor decreases outflow facility. *Invest Ophthalmol Vis Sci*. 2013; 54: 6655–6661. 24030458 10.1167/iovs.13-12766 PMC3796938

[27] Sniegowski M, Mandava N, Kahook MY. Sustained intraocular pressure elevation after intravitreal injection of bevacizumab and ranibizumab associated with trabeculitis. *Open Ophthalmol J*. 2010; 4: 28–29. 20871754 10.2174/1874364101004010028 PMC2944993

[28] Good TJ, Kimura AE, Mandava N, Kahook MY. Sustained elevation of intraocular pressure after intravitreal injections of anti-VEGF agents. *Br J Ophthalmol*. 2011; 95: 1111–1114. 20702430 10.1136/bjo.2010.180729

[29] Hoang QV, Mendonca LS, Della Torre KE, Effect on intraocular pressure in patients receiving unilateral intravitreal anti-vascular endothelial growth factor injections. *Ophthalmology*. 2012; 119: 321–326. 22054994 10.1016/j.ophtha.2011.08.011

[30] Hoang QV, Tsuang AJ, Gelman R, Clinical predictors of sustained intraocular pressure elevation due to intravitreal anti-vascular endothelial growth factor therapy. *Retina*. 2013; 33: 179–187. 22990314 10.1097/IAE.0b013e318261a6f7

[31] Loukianou E, Brouzas D, Apostolopoulos M. Sustained ocular hypertension following intravitreal injections of 0.5 mg/0.05 ml ranibizumab. *Int Ophthalmol*. 2011; 31: 211–213. 21611879 10.1007/s10792-010-9410-z

[32] Mathalone N, Arodi-Golan A, Sar S, Sustained elevation of intraocular pressure after intravitreal injections of bevacizumab in eyes with neovascular age-related macular degeneration. *Graefes Arch Clin Exp Ophthalmol*. 2012; 250: 1435–1440. 22434210 10.1007/s00417-012-1981-0

[33] Segal O, Ferencz JR, Cohen P, Nemet AY, Nesher R. Persistent elevation of intraocular pressure following intravitreal injection of bevacizumab. *Isr Med Assoc J*. 2013; 15: 352–355. 23943979

[34] Kahook MY, Ammar DA. In vitro effects of antivascular endothelial growth factors on cultured human trabecular meshwork cells. *J Glaucoma*. 2010; 19: 437–441. 20164801 10.1097/IJG.0b013e3181ca74de

[35] Liu L, Ammar DA, Ross LA, Silicone oil microdroplets and protein aggregates in repackaged bevacizumab and ranibizumab: effects of long-term storage and product mishandling. *Invest Ophthalmol Vis Sci*. 2011; 52: 1023–1034. 21051703 10.1167/iovs.10-6431 PMC3053093

[36] Yannuzzi NA, Patel SN, Bhavsar KV, Sugiguchi F, Freund KB. Predictors of sustained intraocular pressure elevation in eyes receiving intravitreal anti-vascular endothelial growth factor therapy. *Am J Ophthalmol*. 2014; 158: 319–327. 24814167 10.1016/j.ajo.2014.04.029

[37] Wen JC, Reina-Torres E, Sherwood JM, Intravitreal anti-VEGF injections reduce aqueous outflow facility in patients with neovascular age-related macular degeneration. *Invest Ophthalmol Vis Sci*. 2017; 58: 1893–1898. 28358961 10.1167/iovs.16-20786 PMC6022414

[38] Smith RS, Zabaleta A, Savinova OV, John SW. The mouse anterior chamber angle and trabecular meshwork develop without cell death. *BMC Dev Biol*. 2001; 1: 3. 11228591 10.1186/1471-213X-1-3 PMC31337

[39] Overby DR, Bertrand J, Schicht M, The structure of the trabecular meshwork, its connections to the ciliary muscle, and the effect of pilocarpine on outflow facility in mice. *Invest Ophthalmol Vis Sci*. 2014; 55: 3727–3736. 24833737 10.1167/iovs.13-13699 PMC4059081

[40] Millar JC, Clark AF, Pang IH. Assessment of aqueous humor dynamics in the mouse by a novel method of constant-flow infusion. *Invest Ophthalmol Vis Sci*. 2011; 52: 685–694. 20861483 10.1167/iovs.10-6069

[41] Boussommier-Calleja A, Bertrand J, Woodward DF, Pharmacologic manipulation of conventional outflow facility in ex vivo mouse eyes. *Invest Ophthalmol Vis Sci*. 2012; 53: 5838–5845. 22807298 10.1167/iovs.12-9923 PMC3428113

[42] Overby DR, Bertrand J, Tektas OY, Ultrastructural changes associated with dexamethasone-induced ocular hypertension in mice. *Invest Ophthalmol Vis Sci*. 2014; 55: 4922–4933. 25028360 10.1167/iovs.14-14429 PMC4126794

[43] Li G, Farsiu S, Chiu SJ, Pilocarpine-induced dilation of Schlemm's canal and prevention of lumen collapse at elevated intraocular pressures in living mice visualized by OCT. *Invest Ophthalmol Vis Sci*. 2014; 55: 3737–3746. 24595384 10.1167/iovs.13-13700 PMC4062397

[44] Li P, Shen TT, Johnstone M, Wang RK. Pulsatile motion of the trabecular meshwork in healthy human subjects quantified by phase-sensitive optical coherence tomography. *Biomed Opt Express*. 2013; 4: 2051–2065. 24156063 10.1364/BOE.4.002051 PMC3799665

[45] Sherwood JM, Reina-Torres E, Bertrand JA, Rowe B, Overby DR. Measurement of outflow facility using iPerfusion. *PLoS One*. 2016; 11: e0150694. 26949939 10.1371/journal.pone.0150694 PMC4780770

[46] Bates DO, Cui TG, Doughty JM, VEGF_165_b, an inhibitory splice variant of vascular endothelial growth factor, is down-regulated in renal cell carcinoma. *Cancer Res*. 2002; 62: 4123–4131. 12124351

[47] Stamer WD, Seftor RE, Williams SK, Samaha HA, Snyder RW. Isolation and culture of human trabecular meshwork cells by extracellular matrix digestion. *Curr Eye Res*. 1995; 14: 611–617. 7587308 10.3109/02713689508998409

[48] Coleman DJ, Trokel S. Direct-recorded intraocular pressure variations in a human subject. *Arch Ophthalmol*. 1969; 82: 637–640. 5357713 10.1001/archopht.1969.00990020633011

[49] Last JA, Pan T, Ding Y, Elastic modulus determination of normal and glaucomatous human trabecular meshwork. *Invest Ophthalmol Vis Sci*. 2011; 52: 2147–2152. 21220561 10.1167/iovs.10-6342 PMC3080174

[50] Ferrara N, Gerber HP, LeCouter J. The biology of VEGF and its receptors. *Nat Med*. 2003; 9: 669–676. 12778165 10.1038/nm0603-669

[51] Fong TA, Shawver LK, Sun L, SU5416 is a potent and selective inhibitor of the vascular endothelial growth factor receptor (Flk-1/KDR) that inhibits tyrosine kinase catalysis, tumor vascularization, and growth of multiple tumor types. *Cancer Res*. 1999; 59: 99–106. 9892193

[52] Itokawa T, Nokihara H, Nishioka Y, Antiangiogenic effect by SU5416 is partly attributable to inhibition of Flt-1 receptor signaling. *Mol Cancer Ther*. 2002; 1: 295–302. 12489845

[53] Tille JC, Wang X, Lipson KE, Vascular endothelial growth factor (VEGF) receptor-2 signaling mediates VEGF-C(deltaNdeltaC)- and VEGF-A-induced angiogenesis in vitro. *Exp Cell Res*. 2003; 285: 286–298. 12706123 10.1016/s0014-4827(03)00053-3

[54] Kubo K, Shimizu T, Ohyama S, Novel potent orally active selective VEGFR-2 tyrosine kinase inhibitors: synthesis, structure-activity relationships, and antitumor activities of N-phenyl-N′-{4-(4-quinolyloxy)phenyl}ureas. *J MedChem*. 2005; 48: 1359–1366. 10.1021/jm030427r 15743179

[55] Woolard J, Wang WY, Bevan HS, VEGF_165_b, an inhibitory vascular endothelial growth factor splice variant: mechanism of action, in vivo effect on angiogenesis and endogenous protein expression. *Cancer Res*. 2004; 64: 7822–7835. 15520188 10.1158/0008-5472.CAN-04-0934

[56] Qiu Y, Ferguson J, Oltean S, Overexpression of VEGF_165_b in podocytes reduces glomerular permeability. *J Am Soc Nephrol*. 2010; 21: 1498–1509. 20688932 10.1681/ASN.2009060617 PMC3013528

[57] Peiris-Pagès M. The role of VEGF_165_b in pathophysiology. *Cell Adh Migr*. 2012; 6: 561–568. 23076130 10.4161/cam.22439 PMC3547904

[58] Achen MG, Jeltsch M, Kukk E, Vascular endothelial growth factor D (VEGF-D) is a ligand for the tyrosine kinases VEGF receptor 2 (Flk1) and VEGF receptor 3 (Flt4). *Proc Natl Acad Sci U S A*. 1998; 95: 548–553. 9435229 10.1073/pnas.95.2.548 PMC18457

[59] Achen MG, Stacker SA. The vascular endothelial growth factor family; proteins which guide the development of the vasculature. *Int J Exp Pathol*. 1998; 79: 255–265. 10193309 10.1046/j.1365-2613.1998.700404.x PMC3220209

[60] Yu L, Wu X, Cheng Z, Interaction between bevacizumab and murine VEGF-A: a reassessment. *Invest Ophthalmol Vis Sci*. 2008; 49: 522–527. 18234994 10.1167/iovs.07-1175

[61] Miki K, Miki A, Matsuoka M, Effects of intraocular ranibizumab and bevacizumab in transgenic mice expressing human vascular endothelial growth factor. *Ophthalmology*. 2009; 116: 1748–1754. 19643496 10.1016/j.ophtha.2009.05.020 PMC2913289

[62] Roberts WG, Palade GE. Increased microvascular permeability and endothelial fenestration induced by vascular endothelial growth factor. *J Cell Sci*. 1995; 108: 2369–2379. 7673356 10.1242/jcs.108.6.2369

[63] Breier G, Albrecht U, Sterrer S, Risau W. Expression of vascular endothelial growth factor during embryonic angiogenesis and endothelial cell differentiation. *Development*. 1992; 114: 521–532. 1592003 10.1242/dev.114.2.521

[64] Ford KM, Saint-Geniez M, Walshe TE, D'Amore PA. Expression and role of VEGF-A in the ciliary body. *Invest Ophthalmol Vis Sci*. 2012; 53: 7520–7527. 23081980 10.1167/iovs.12-10098 PMC3493183

[65] Tamm ER. The trabecular meshwork outflow pathways: structural and functional aspects. *Exp Eye Res*. 2009; 88: 648–655. 19239914 10.1016/j.exer.2009.02.007

[66] Hillman NJ, Whittles CE, Pocock TM, Williams B, Bates DO. Differential effects of vascular endothelial growth factor-C and placental growth factor-1 on the hydraulic conductivity of frog mesenteric capillaries. *J Vasc Res*. 2001; 38: 176–186. 11316953 10.1159/000051044

[67] Whittles CE, Pocock TM, Wedge SR, ZM323881, a novel inhibitor of vascular endothelial growth factor-receptor-2 tyrosine kinase activity. *Microcirculation*. 2002; 9: 513–522. 12483548 10.1038/sj.mn.7800164

[68] Olofsson B, Jeltsch M, Eriksson U, Alitalo K. Current biology of VEGF-B and VEGF-C. *Curr Opin Biotechnol*. 1999; 10: 528–535. 10600689 10.1016/s0958-1669(99)00024-5

[69] Hamanaka T, Bill A, Ichinohasama R, Ishida T. Aspects of the development of Schlemm's canal. *Exp Eye Res*. 1992; 55: 479–488. 1426078 10.1016/0014-4835(92)90121-8

[70] Simons M, Gordon E, Claesson-Welsh L. Mechanisms and regulation of endothelial VEGF receptor signalling. *Nat Rev Mol Cell Biol*. 2016; 17: 611–625. 27461391 10.1038/nrm.2016.87

[71] Karnezis T, Shayan R, Caesar C, VEGF-D promotes tumor metastasis by regulating prostaglandins produced by the collecting lymphatic endothelium. *Cancer Cell*. 2012; 21: 181–195. 22340592 10.1016/j.ccr.2011.12.026

[72] Winkler NS, Fautsch MP. Effects of prostaglandin analogues on aqueous humor outflow pathways. *J Ocul Pharmacol Ther*. 2014; 30: 102–109. 24359106 10.1089/jop.2013.0179 PMC3991965

[73] Millard LH, Woodward DF, Stamer WD. The role of the prostaglandin EP4 receptor in the regulation of human outflow facility. *Invest Ophthalmol Vis Sci*. 2011; 52: 3506–3513. 21245402 10.1167/iovs.10-6510

[74] Kim YW, Byzova TV. Oxidative stress in angiogenesis and vascular disease. *Blood*. 2014; 123: 625–631. 24300855 10.1182/blood-2013-09-512749 PMC3907751

[75] Galvagni F, Pennacchini S, Salameh A, Endothelial cell adhesion to the extracellular matrix induces c-Src-dependent VEGFR-3 phosphorylation without the activation of the receptor intrinsic kinase activity. *Circ Res*. 2010; 106: 1839–1848. 20431062 10.1161/CIRCRESAHA.109.206326

[76] Downs JC, Burgoyne CF, Seigfreid WP, 24-hour IOP telemetry in the nonhuman primate: implant system performance and initial characterization of IOP at multiple timescales. *Invest Ophthalmol Vis Sci*. 2011; 52: 7365–7375. 21791586 10.1167/iovs.11-7955 PMC3183973

[77] Rohen JW, Futa R, Lütjen-Drecoll E. The fine-structure of the cribriform meshwork in normal and glaucomatous eyes as seen in tangential sections. *Invest Ophthalmol Vis Sci*. 1981; 21: 574–585. 7287347

[78] Bradley JM, Kelley MJ, Zhu X, Effects of mechanical stretching on trabecular matrix metalloproteinases. *Invest Ophthalmol Vis Sci*. 2001; 42: 1505–1513. 11381054

[79] Li A, Leung CT, Peterson-Yantorno K, Stamer WD, Civan MM. Cytoskeletal dependence of adenosine triphosphate release by human trabecular meshwork cells. *Invest Ophthalmol Vis Sci*. 2011; 52: 7996–8005. 21896846 10.1167/iovs.11-8170 PMC3220413

[80] Tripathi RC, Li JP, Tripathi BJ, Chalam KV, Adamis AP. Increased level of vascular endothelial growth factor in aqueous humor of patients with neovascular glaucoma. *Ophthalmology*. 1998; 105: 232–237. 9479280 10.1016/s0161-6420(98)92782-8

[81] Hu DN, Ritch R, Liebmann J, Vascular endothelial growth factor is increased in aqueous humor of glaucomatous eyes. *J Glaucoma*. 2002; 11: 406–410. 12362079 10.1097/00061198-200210000-00006

[82] Tong JP, Chan WM, Liu DT, Aqueous humor levels of vascular endothelial growth factor and pigment epithelium-derived factor in polypoidal choroidal vasculopathy and choroidal neovascularization. *Am J Ophthalmol*. 2006; 141: 456–462. 16490490 10.1016/j.ajo.2005.10.012

[83] Sawada O, Kawamura H, Kakinoki M, Sawada T, Ohji M. Vascular endothelial growth factor in aqueous humor before and after intravitreal injection of bevacizumab in eyes with diabetic retinopathy. *Arch Ophthalmol*. 2007; 125: 1363–1366. 17923544 10.1001/archopht.125.10.1363

[84] Roh MI, Kim HS, Song JH, Concentration of cytokines in the aqueous humor of patients with naive, recurrent and regressed CNV associated with amd after bevacizumab treatment. *Retina*. 2009; 29: 523–529. 19262441 10.1097/IAE.0b013e318195cb15

[85] Takai Y, Tanito M, Ohira A. Multiplex cytokine analysis of aqueous humor in eyes with primary open-angle glaucoma, exfoliation glaucoma, and cataract. *Invest Ophthalmol Vis Sci*. 2012; 53: 241–247. 22159018 10.1167/iovs.11-8434

[86] Costagliola C, Daniele A, dell'Omo R, et al. Aqueous humor levels of vascular endothelial growth factor and adiponectin in patients with type 2 diabetes before and after intravitreal bevacizumab injection. *Exp Eye Res*. 2013; 110: 50–54. 23454098 10.1016/j.exer.2013.02.004

[87] Hsu MY, Yang CY, Hsu WH, Monitoring the VEGF level in aqueous humor of patients with ophthalmologically relevant diseases via ultrahigh sensitive paper-based ELISA. *Biomaterials*. 2014; 35: 3729–3735. 24484673 10.1016/j.biomaterials.2014.01.030

[88] Wang JW, Zhou MW, Zhang X, Short-term effect of intravitreal ranibizumab on intraocular concentrations of vascular endothelial growth factor-A and pigment epithelium-derived factor in neovascular glaucoma. *Clin Experiment Ophthalmol*. 2015; 43: 415–421. 25488632 10.1111/ceo.12477

[89] Liu XH, Kirschenbaum A, Lu M, Prostaglandin E2 induces hypoxia-inducible factor-1alpha stabilization and nuclear localization in a human prostate cancer cell line. *J Biol Chem*. 2002; 277: 50081–50086. 12401798 10.1074/jbc.M201095200

[90] Wu WK, Llewellyn OP, Bates DO, Nicholson LB, Dick AD. IL-10 regulation of macrophage VEGF production is dependent on macrophage polarisation and hypoxia. *Immunobiology*. 2010; 215: 796–803. 20692534 10.1016/j.imbio.2010.05.025

[91] McMenamin PG, Holthouse I. Immunohistochemical characterization of dendritic cells and macrophages in the aqueous outflow pathways of the rat eye. *Exp Eye Res*. 1992; 55: 315–324. 1426064 10.1016/0014-4835(92)90196-y

[92] Flügel C, Kinne RW, Streilein JW, Lütjen-Drecoll E. Distinctive distribution of HLA class II presenting and bone marrow derived cells in the anterior segment of human eyes. *Curr Eye Res*. 1992; 11: 1173–1183. 1283365 10.3109/02713689208999542

[93] Choi DY, Ortube MC, McCannel CA, Sustained elevated intraocular pressures after intravitreal injection of bevacizumab, ranibizumab, and pegaptanib. *Retina*. 2011; 31: 1028–1035. 21836409 10.1097/IAE.0b013e318217ffde

[94] Kahook MY, Liu L, Ruzycki P, High-molecular-weight aggregates in repackaged bevacizumab. *Retina*. 2010; 30: 887–892. 20458261 10.1097/IAE.0b013e3181d50cea

[95] Gal-Or O, Dotan A, Dachbash M, Bevacizumab clearance through the iridocorneal angle following intravitreal injection in a rat model. *Exp Eye Res*. 2016; 145: 412–416. 26923799 10.1016/j.exer.2016.02.006

