# Supplementary material for: VEGF as a Paracrine Regulator of Conventional Outflow Facility
Source: Invest Ophthalmol Vis Sci. 2017 Mar;58(3):1899–908. doi: 10.1167/iovs.16-20779 (PMC5374885; doi:10.1167/iovs.16-20779)
Supplement: Supplement 1 [file iovs-58-03-03_s01.docx]

**Supplemental Figure 1:** *VEGF-A labeling in the murine TM visualized using a second anti-VEGF antibody.* Immunohistochemistry of the limbus in C57BL/6 mice showing the localization of VEGF-A_164_ (A: green, AF-493, R&D Biosystems), CD31/PECAM-1 (B: red), and the merged images (C). The inset to panel C shows a bright field image of the same section shown in panels A and B. VEGF-A_164_ is present within the trabecular meshwork (TM, arrowheads) near Schlemm’s canal that expresses CD31. VEGF-A_164_ is also present in the ciliary epithelium and surrounding CD31 positive vessels in the ciliary body (CB). Negative control images were prepared and imaged except omitting the primary antibodies for VEGF-A_164_ (D) and CD31 (E). The merged images from panels D and E are superimposed on the bright field image (F). Methods used to acquire these images are described below. Asterisks indicate the lumen of Schlemm’s canal. Scale bars are 200 µm. Co: cornea.

**
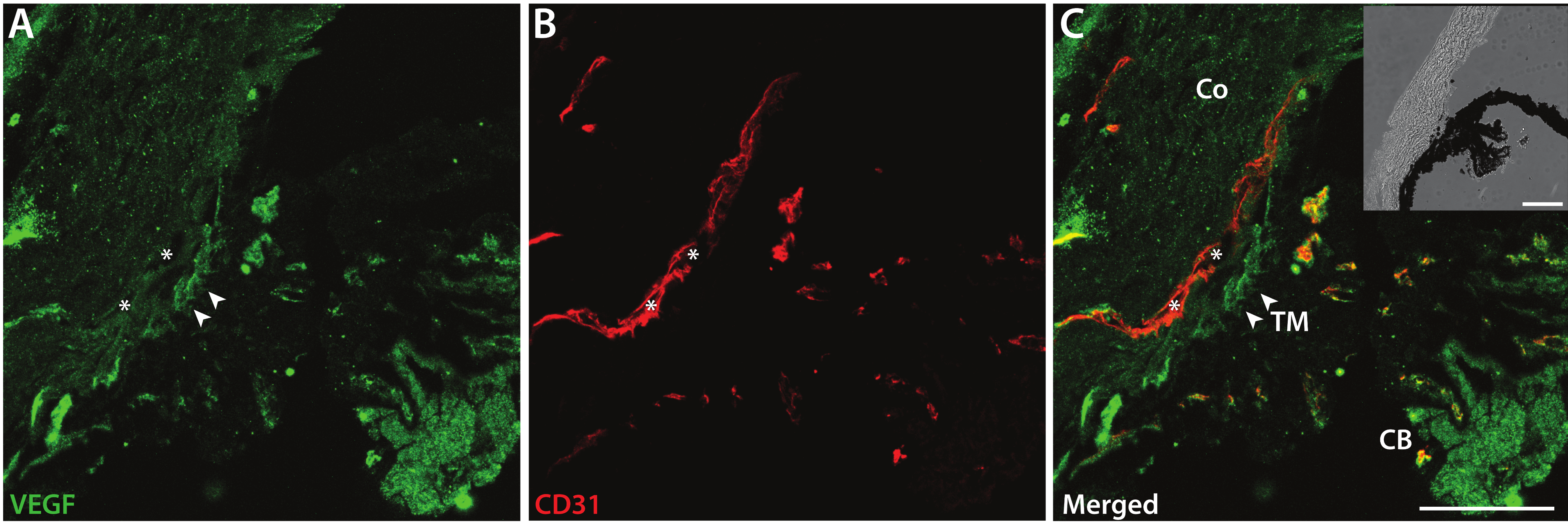
**

E

F

D

**Methods for Supplemental Figure 1**

Freshly enucleated eyes (*n* = 3) from 3 C57BL/6 mice aged 9 to 13 weeks were fixed in 4% formaldehyde in PBS for 2 hours followed by multiple washes in PBS. Eyes were cleared of extraocular tissues and hemisected at the equator, carefully removing the lens. The anterior segment was submerged in 20% sucrose in PBS overnight at 4ºC, and then embedded in a 1:1 mixture of 20% sucrose solution and embedding compound (Tissue-Tek OCT; Sakura-Finetek, Torrance, CA). Cryosections were cut to 16 µm through the limbus using a Cryostat OTF/AS (Bright Instrument Co Ltd, UK), placed on a microscope adhesion slide (SUPERFROST^®^, Electron Microscopy Sciences, Hatfield PA) and allowed to air dry for 30 minutes. Samples were stored at ‑20ºC until used. Samples were allowed to equilibrate at room temperature for 1 hour, then permeabilized and blocked with 10% horse serum (Stratech Scientific Ltd., Newmarket UK) and 1% Triton-X in PBS solution for 30 minutes. Samples were incubated with polyclonal goat anti-mouse VEGF-A_164_ (polyclonal, AF-493, R&D Systems, Minneapolis, MN) diluted to 15 µg/ml in blocking solution for 2 hours at room temperature. Samples were then washed three times in PBS for 5 minutes each and incubated with donkey secondary antibodies against goat IgG conjugated to Alexa Fluor^®^ 488 (Life Technologies, Waltham, MA) diluted 1:200 in blocking solution for 1 hour at room temperature. Samples were then washed in PBS and incubated with rat primary antibodies against mouse CD31/PECAM1 (Clone MEC 13.3, BD Biosciences, San Jose, CA) diluted at 1:100 in blocking solution for 2 hours at room temperature. Samples were washed in PBS and then incubated with goat secondary antibodies against rat IgG conjugated to Alexa Fluor 594^®^ (Life Technologies, Waltham, MA) diluted 1:200 in blocking buffer for 1 hour at room temperature. Samples were washed in PBS (3x) and then mounted on a glass microscope slide using fluorescent mounting medium (ProLong^®^ Diamond Antifade Mountant, Life Technologies, Waltham, MA) and allowed to dry overnight at 4ºC. Samples were imaged on a Leica SP5 confocal microscope (Leica, Milton Keynes, UK) at 20x magnification. Negative controls were processed identically except omitting the primary antibodies.


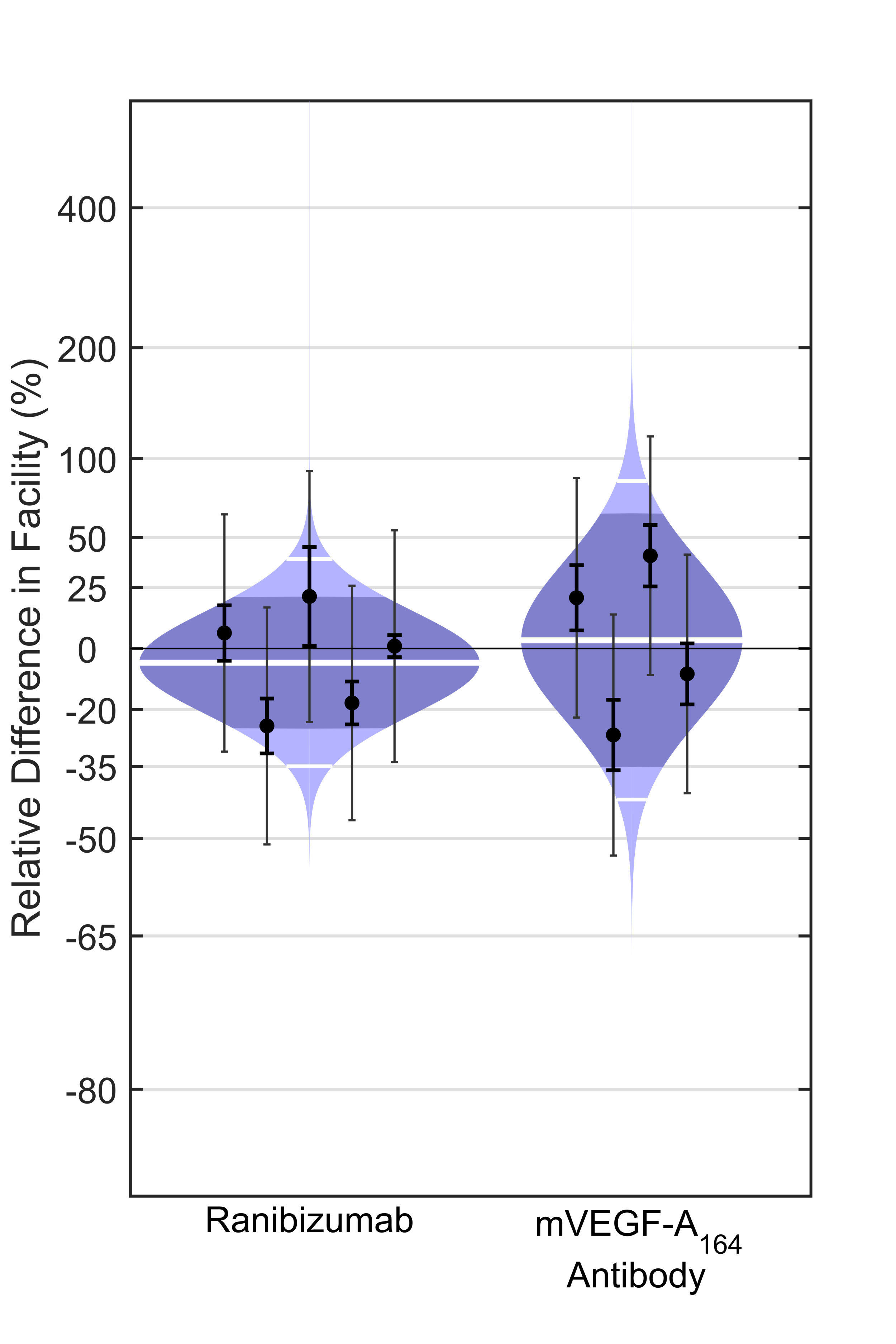
**Supplemental Figure 2:** *Acute exposure to anti-VEGF antibodies does not significantly affect outflow facility in enucleated mouse eyes*. Cello plots showing the relative difference in outflow facility between treated eyes of C57BL/6 mice perfused with anti-VEGF antibodies versus contralateral eyes perfused with vehicle alone for 0.14 mg/ml ranibizumab (Novartis, Basel, Switzerland) or 0.14 mg/ml of polyclonal antibody against mouse VEGF-A_164_ (AF-493, R&D Biosystems, Minneapolis, MN). Neither ranibizumab nor anti-murine VEGF-A_164_ appeared to significantly affect outflow facility over relatively short time scales (corresponding to ~2 hour duration of perfusion) with an average difference of -5% (*p* = 0.58, *n* = 5, weighted *t*-test) and 3% (*p* = 0.85, *n* = 4, weighted *t*-test), respectively. Data points represent the relative facility difference of a treated eye with respect to its contralateral untreated eye for individual pairs. The thick white lines represent the geometric means of the relative differences for each group. The remaining symbols are as defined in Figure 2 of the main text.
